# The impact of clean energy demonstration province policies on carbon intensity in Chinese counties based on the multi-phase PSM-DID method

**DOI:** 10.1007/s11356-023-31799-5

**Published:** 2024-01-18

**Authors:** Lei Chen, Cunjing Liu, Zhezhou Li, Difan Wu

**Affiliations:** 1 Rural Development Institute, Yan’an University, Yan’an, 716000 China; 2 School of Economics and Management, Yan’an University, Yan’an, 716000 China; 3 School of Economics and Management, Fuzhou University of International Studies and Trade, Fuzhou, 350202 China; 4 State Grid Shanghai Electric Power Company Shibei Power Supply Company, Shanghai, 200070 China

**Keywords:** Carbon intensity, Carbon emissions, Economic development level, PSM-DID, County-level of China

## Abstract

Based on China’s empirical data from 2000 to 2020 of 1875 county-level administrative units, combined with the multi-phase by the propensity score matching and difference-in-difference (PSM-DID) model, this paper studies the impact of clean energy demonstration province policies on the carbon intensity of pilot counties, and its further impact on carbon emissions and economic development level. The results showed that 1. from a county-level perspective, although the economic development level of the pilot areas of clean energy demonstration provinces has improved as the carbon emissions have also increased, what is more, the carbon intensity has also significantly improved in this process; 2. there is no time lag in the impact of policies on the carbon intensity of counties, and the impact effects gradually increase over time along with strong regional heterogeneity; 3. the clean energy demonstration policy has weakened the technological level of the county and reduced the proportion of industrial-added value to GDP, thereby increasing the carbon intensity of the county through these intermediaries.

## Introduction

In order to promote the development of sustainable energy, promote the adjustment of energy consumption structure, reduce environmental pollution, improve air quality, and achieve sustainable economic development, China implemented the clean energy demonstration provincial policy. To be specific, Ningxia was included in the clean energy demonstration province policy in 2012; Zhejiang in 2014; Sichuan, Tibet, and Gansu in 2016; Qinghai in 2018 (see Appendix 1), which mainly focuses on the comprehensive demonstration base of clean energy, the innovation base of clean energy technology, and the practice base of energy system and mechanism reform to reduce dependence on traditional fossil energy, reduce pollution emissions, protect the environment, and promote sustainable development (Zhou et al. 2023). In the “13th Five Year Plan for Energy Development” issued by the National Energy Administration of China in December, 2016, the construction of clean energy demonstration provinces was included as a key project in the energy consumption revolution. China’s “2035 Long Range Goals Outline” and “14th Five Year Plan for Renewable Energy Development” and other documents start from the top-level design and follow up with local plans to build a comprehensive clean energy policy system to ensure energy supply and reasonable low-carbon consumption. In accordance with the planning concept, the pilot areas have formulated their specific work plans and put forward the need to “steadily promote the revolution of the energy system.” Speeding up the building of a clean, low-carbon, safe, and efficient energy system, promoting the replacement of old and new driving forces, and promoting high-quality economic development have become crucial green development issues in the current clean energy demonstration provincial policy pilot areas of China. Carbon intensity is an indicator that measures the costs associated with carbon emissions in economic growth, reflecting the concept of achieving coordinated development between economic growth and reducing carbon emissions. Reducing carbon intensity can promote the low-carbon and green transformation of the economy and society. At the same time, it is also necessary for the industrial economy to move toward sustainable development.

Currently, many scholars have paid attention to the policy effects surrounding China’s green economy and clean energy. China’s low-carbon macro policy, including those aimed at improving energy efficiency, and applying renewable energy are being widely applied in the form of command-mandatory tools, market-economic tools, and voluntary tools (Wang et al. 2015). Most studies on the policy effects of green economy and clean energy in China focus on the panel data at the provincial and municipal levels. Cheng et al. (2019) finds that low-carbon pilot policy in China has a significant inhibitory effect on the carbon emission intensity of pilot cities, and the policy impact effect gradually increases with the increase of pilot years (Cheng et al. 2019). Yang et al. (2022) found that the new energy demonstration city policy can significantly improve the green total factor productivity of resource-based cities (Yang et al. 2022). Razzaq and Yang (2023) found that CIEP decreased carbon emissions intensity by improving energy utilization efficiency and intensifying local government environmental attention (Razzaq and Yang 2023).

In addition to the above research on municipal green development policies based on municipal panel data, many scholars also conducted research on some provincial policies in China through provincial panel data. Based on provincial panel data and combined with the difference-in-difference (DID) method, Zhou et al. (2023) found that the policies of clean energy demonstration provinces improved the level of economic development while significantly inhibiting carbon emissions in pilot areas, achieving double dividends of environment and economy (Zhou et al. 2023). Based on the panel data of 26 provinces in China from 2000 to 2017, Hu et al. (2023) used the carbon dioxide emission efficiency index to predict the carbon intensity of the national ecological civilization pilot zone in China by national ecological policy by the combination of the auto-regressive integrated moving average model and back-propagation neural network model (Hu et al. 2023).

Although many scholars have studied China’s green development policies at the macro level through various methods, few scholars have examined the policy effects of policies of the macro level (city or provincial level) from the micro perspective (county-level administrative units). In particular, for a provincial level policy such as the clean energy demonstration province, the performance evaluation based on the overall performance at the provincial level may seriously ignore the performance of micro individuals, making the evaluation of the policy effect incomplete and one dimensional.

Paying attention to county-level carbon intensity data is more critical than provincial-level data for the following reasons. (1) More specific and accurate: county-level administrative divisions are more microscopic than provinces, reflecting more specific regional characteristics and economic development status, so county-level carbon intensity data is more precise and reliable. (2) A more intuitive assessment of the current situation: for formulating emission reduction plans and implementing policies, it is necessary to have a detailed understanding of the carbon intensity in different regions. Only by comparing county-level carbon intensity data can the local emission reduction achievements and situation be more intuitively evaluated. (3) More refined planning and regulation: focusing on county-level carbon intensity data can help the government better analyze the local industrial structure and energy consumption characteristics, formulate more refined planning and emission reduction measures, and promote the deep promotion of low-carbon development.

Can the implementation of clean energy demonstration province policies effectively reduce carbon intensity in pilot county areas which means controlling carbon emissions and improving their economic development level? It is a critical issue that should be studied and solved, so a comprehensive evaluation of the impact of clean energy demonstration province policies has essential academic guidance and practical significance for further precise promotion and improvement of demonstration province policies.

The marginal contributions of this paper mainly focus on the following aspects: first, based on the propensity score matching and difference-in-difference (PSM-DID) model, this paper profoundly explores the impacts of clean energy demonstration provinces on the carbon intensity of the counties covered by the policy and its mechanism, besides, further analyze their impacts on carbon emissions and economic development level. Secondly, heterogeneity analysis was carried out according to Hu line to enrich further the policy effectiveness analysis of clean energy demonstration provinces based on regional heterogeneity. Finally, based on the study of the general connotation law of the clean energy demonstration provincial policy, this paper provides a valuable reference for further promoting energy conservation and emission reduction and high-quality development.

## Theoretical analysis and research hypotheses

### The effect of clean energy demonstration provincial policy on county level carbon intensity

Based on China’s provincial panel data can achieve double dividends of environment and economy, and improve the level of economic development while curbing carbon emissions in pilot areas (Zhou et al. 2023). However, the performance of provincial data may mask the heterogeneity of the more micro county dimension. The policy of clean energy demonstration province can cultivate major government projects and promote preferential policies in the clean energy industry (Yang et al. 2021; Chen et al. 2020a), which can simultaneously promote the overall economic stability and development in both the pilot provinces and the regions they represent without doubt. Although the pilot provinces are committed to building clean energy demonstration zones, due to limited resources and weak industrial base, the policy may have an opposite policy effect on county carbon emissions.

According to the resource dependence theory, because some resource providers have relatively more resources and others have relatively less, it is easier for them to obtain more resources, thus forming a circular process, resulting in uneven resource distribution. Urban areas or economically developed areas tend to concentrate more resources, including human resources, financial resources, and technical resources. This enables the implementation of the clean energy model provincial policy to receive better support and input, thereby improving the effect of the policy. In contrast, the human resources, financial resources, and technical resources of county-level administrative units except urban areas are relatively limited, the carbon dioxide emission marginal reduction cost (Zhao and Xi 2022) is heterogeneous among county-level administrative units (Duan et al. 2018; Wang and He 2017). This leads to the difficulty and poor effect of the implementation of clean energy demonstration provincial policies in county level.

Besides, due to the weak industrial base, the clean energy model provincial policy is a burden rather than a positive incentive for those enterprises located in county-level administrative units. Given the relatively rough industry and weak economic development foundation of counties, as well as the lag of industrial structure changes (Dong et al. 2018), the green economy is likely to be a huge challenge for enterprise at the county level. The allocation of green-related assets may increase the investment costs of county-level enterprises in the short term, affecting their short-term economic benefits (Li and Gao 2018); county-level enterprises with limited comprehensive ability are more likely to choose “pollution first, treatment later.” In addition, the county industry itself is not conducive to saving land nor can it give play to the externality of industrial agglomeration (Fan et al. 2021; Tian et al. 2019), which tends to correlate positively with energy efficiency. Moreover, industrial structure upgrading has a significant negative spatial correlation with carbon dioxide emissions (Zhao et al. 2022), which reduces carbon dioxide emissions by improving energy efficiency. However, the county-level industrial structure is often locked at the bottom of the industrial chain, with low added value and difficult to change.

- **Hypothesis 1**. The implementation of China’s clean energy demonstration provincial policy will enhance the carbon intensity of pilot counties.
- **Hypothesis 2**. The implementation of the clean energy demonstration provincial policy will promote county-level economic development and increase carbon emissions at the same time.

### The mediating effect of technical progress on county level carbon intensity

Innovation can drive the development and application of clean energy sources, such as wind, solar, and geothermal energy. Compared with traditional fossil fuels, the use of clean energy can significantly reduce carbon emission intensity. Innovation in renewable energy technology helps reduce carbon dioxide emissions (Wang and Zhu 2020; Lin and Zhu 2019; Su et al. 2021). Ecological innovation and green investments can synergy, green investment can provide financial support and market demand for ecological innovation, and ecological innovation also provides more choices and investment opportunities for green investment, this synergic effect can reduce the carbon dioxide emissions (Temesgen Hordofa et al. 2023) including the indirect carbon dioxide emissions directly generated by enterprises and related value chains (Qureshi et al. 2022).

Moreover, innovation can help us develop and apply technologies and methods that use resources more efficiently. By optimizing the production process, reducing energy consumption, and reducing material consumption, the carbon emissions required for each unit of product can be reduced, thereby reducing carbon intensity. There is a significant negative correlation between technological progress and industrial carbon intensity (Hu et al. 2023; Pan et al. 2021; Albitar et al. 2023; Dauda et al. 2019), and energy efficiency and reduced environmental innovation will reduce China’s carbon dioxide emissions in the long run (Li et al. 2022a, b).

Green innovations induce stronger reductions in carbon dioxide emissions when policy quality is high (Yuan et al. 2022). The county-level administrative units except urban areas may have deficiencies in the design and implementation of innovation-related policies, resulting in significant differences in the implementation effects of clean energy demonstration provinces. This may lead to the fact that in some county-level administrative units, clean energy model provincial policies may not only promote innovation but also actually inhibit innovation.

- **Hypothesis 3**. Technological progress plays a significant mediating effect on the policy effect of clean energy demonstration province to improve county carbon intensity.

### The mediating effect of upgrading of industrialization on county level carbon intensity

The application of clean energy is gradually expanding with the upgrading of industrial structure, which can reduce greenhouse gas emissions and thus reduce carbon intensity. In addition, with the upgrading of industrial structure, enterprises pay more attention to energy management and improve energy efficiency (Yu et al. 2018). More precisely, the relatively high carbon production structure leads to higher carbon intensity (Lin and Zhu 2019); the industrial carbon intensity showed a significant downward trend by increasing per capita industrial value (Hu et al. 2023). An increase in the share of secondary industry output in GDP would reduce total energy consumption and thus reduce carbon dioxide emissions (Wei and Zhang 2020; Zhao et al. 2020). The improvement of the manufacturing technology level and the relatively low-carbon production structure will promote the low-carbon development mode (Tian et al. 2019; Mi et al. 2015).

Through rational adjustment of industrial structure, energy intensity can be reduced without affecting economic growth. With the promotion of the clean energy demonstration provincial policy, the government will gradually increase the punitive policy for high-polluting low-end manufacturing in urban areas, and enterprises will choose to transfer low-end industries to non-urban county-level administrative units with relatively lose control in order to reduce costs. In addition, the siphon phenomenon in the urban area will lead to the scarcity of resources in non-urban county-level administrative units, that is, the urban area has a strong attraction, and the population, resources, and funds tend to gather in the urban center. Due to the limited resources, the development of counties may receive insufficient policy support, resulting in a relative lag in industrial upgrading.

- **Hypothesis 4**. Upgrading of industrialization plays a significant mediating effect on the policy effect of clean energy demonstration province to improve county carbon intensity.

## Methodology and indicator

### Model specification

In order to scientifically evaluate the impacts of clean energy demonstration provincial policies on county carbon intensity in pilot areas, a multi-phase PSM-DID model was used in this paper (Zhou et al. 2023), which can reduce “selective bias” and help alleviate endogenous problems. Compared with the traditional difference model or the simple differential model, the multi-period differential model takes into account the effects of multiple periods and provides a more comprehensive analysis and more accurate estimation of more complex economic phenomena. This approach is essentially a statistic of all possible two-group or two-phase DID average treatment effect on the treated (ATT) based on the empirical data. In this paper, the pilot policy of China’s clean energy demonstration province is regarded as a kind of “quasi-natural experiment” and takes the actual years of implementing clean energy demonstration provincial policies in the counties included in the pilot as the time node of policy intervention. The subjects were divided into a treatment group and a control group to compare the differences in carbon intensity between the two groups before and after the policy implementation. Furthermore, this paper makes an in-depth study of the influence of the economy and carbon emission in the areas covered by the policy. At the same time, a mediation effect model was established to verify the mechanism of the influence of clean energy demonstration province policies on carbon intensity. Specifically, the models shown in this paper are as follows:

1 $${CI}_{i,t}={\alpha }_{0}+{\alpha }_{1}\times {Policy}_{i,t}+\gamma \times {Control}_{i,t}+{\lambda }_{1}+{\mu }_{t}+{\varepsilon }_{i,t}$$

2 $${Y}_{i,t}={\beta }_{0}+{\beta }_{1}\times {Policy}_{i,t}+\gamma \times {Control}_{i,t}+{\lambda }_{1}+{\mu }_{t}+{\varepsilon }_{i,t}$$

3 $${CE}_{i,t}={\chi }_{0}+{\chi }_{1}\times {Policy}_{i,t}+\gamma \times {Control}_{i,t}+{\lambda }_{1}+{\mu }_{t}+{\varepsilon }_{i,t}$$

where ${CI}_{i,t}$ is the explained variable which represents the carbon intensity level of the *i* county in the *t* year in Eq. (1); ${Y}_{i,t}$ is the explained variable which represents the economic development level of the *i* county-level administrative unit in the *t* year in Eq. (2); ${CE}_{i,t}$ is the explained variable which represents the carbon emissions of the *i* county-level administrative unit in the *t* year in Eq. (3); ${Policy}_{i,t}$ is the core explanatory variable which represents the dummy variable of whether the *i* county-level administrative unit is covered by the clean energy demonstration provincial policy in the *t* year, if the county-level administrative unit is covered by the policy in the year, the value is 1, otherwise, it is 0; ${Control}_{i,t}$ is the control variable; ${\lambda }_{1}$ represents entity fixed effects; ${\mu }_{t}$ represents time-fixed effect; ${\varepsilon }_{i,t}$ represents stochastic error term. When ${\alpha }_{1}$ is significantly negative (positive), it indicates that the policy of the clean energy demonstration province has significantly reduced (improved) the carbon intensity of the counties in the pilot area, while keeping other conditions unchanged.

### Indicator selection and data source

#### Explained variable

The explained variable in the baseline regression analysis of this paper is carbon intensity $\left({CI}_{i,t}\right)$, which is the amount of carbon dioxide emitted per unit of GDP. In general, carbon intensity indicators decline with technological progress and economic growth. The explained variables in the further analysis are the economic development level (${Y}_{i,t}$), and the carbon dioxide emissions (${CE}_{i,t}$), referred to Wang and Wang (2020) the calculation formula of carbon intensity is adapted (Wang and Wang 2020).

#### Core explanatory variable

The core explanatory variable of this article is the policy of the clean energy demonstration province (${Policy}_{i,t}$); if the county is covered by the clean energy model provincial policy in the current year, it takes 1 for that year and subsequent years; otherwise, it takes 0.

#### Control variable

In order to further ensure the reliability of the research results, this paper draws on the existing research; urbanization rate (Rate-Ur), the proportion of primary industry in GDP (rate-primary), the proportion of secondary industry in GDP (rate-secondary), the proportion of fiscal revenue in GDP (rate-FR), the proportion of fiscal expenditure in GDP (rate-FE), the degree of fiscal tensions (D-FT), the resident savings rate (rate-RS), the regional credit leverage (L-RC), and the proportion of fixed assets station GDP (rate-FA) are selected as the control variables. The description of specific variables and corresponding descriptive statistics are shown in Table 1.

**Table 1:** Descriptive statistics

Variables	Meaning and assignment	Observation	Mean	STD
Explained variable				
*CI*	Carbon dioxide emissions/regional GDP	32,492	4.5362	18.7104
*CE*	Carbon emissions based on county-level units, in millions of tons	34,452	0.2940	1.3238
*Y*	Gross regional production value based on county administrative units, unit: RMB 100 million	39,371	13.0104	1.2558
Core explanatory variable				
*Policy*	If it is covered by the clean energy demonstration provincial policy, the value is 1; otherwise, it is 0	42,212	0.0441	0.2053
Control variable				
Rate-Ur	The proportion of urban population in total population (including agricultural and non-agricultural)	24,210	0.1712	0.7726
Rate-primary	Primary industry output value/regional GDP	39,156	0.2360	0.1388
Rate-secondary	Secondary industry output value/regional GDP	39,353	0.4091	0.1609
Rate-FR	Local government general budget revenue/regional GDP	34,551	0.0523	0.0357
Rate-FE	General budget expenditure of local finance/regional GDP	34,578	0.2296	0.2488
D-FT	Local government general budget revenue/local government general budget expenditure	35,185	5.3414	7.2928
Rate-RS	Household savings deposit balance at the end of the year/regional GDP	37,714	0.7300	0.5189
L-RC	Balance of all loans of financial institutions at the end of the year/regional GDP	33,552	0.6215	0.4304
Rate-FA	Total social investment in fixed assets/regional GDP	29,542	0.6540	0.4908

Carbon emission and regional GDP are logarithmic

### Sample selection and data sources

The treatment group and control group were determined according to the implementation time and scope of China’s clean energy Demonstration Province construction policy. Given the severe lack of county data in Xizang, Xinjiang, and Inner Mongolia provinces of China, they were excluded from the samples. County panel data from 2000 to 2020 were adopted, and county carbon emission data (Chen et al. 2020a, b) came from Chen et al. (2020b); regional GDP, urbanization rate, regional economic and fiscal data, household savings, loan balance, and fixed asset investment are mainly from the county economic database of CSMAR and the County Statistical yearbook of the National Bureau of Statistics and are supplemented by cross-comparison; the regional GDP of each county was deflated with the base value of 2000; the number of patents published and authorized comes from the State Intellectual Property Office. Missing values were imputed with the use of interpolation.

## Baseline regression analysis and robustness test

### Baseline regression of policy effect analysis on carbon intensity

#### PSM-Mahalanobis distance matching

**Fig. 1:**
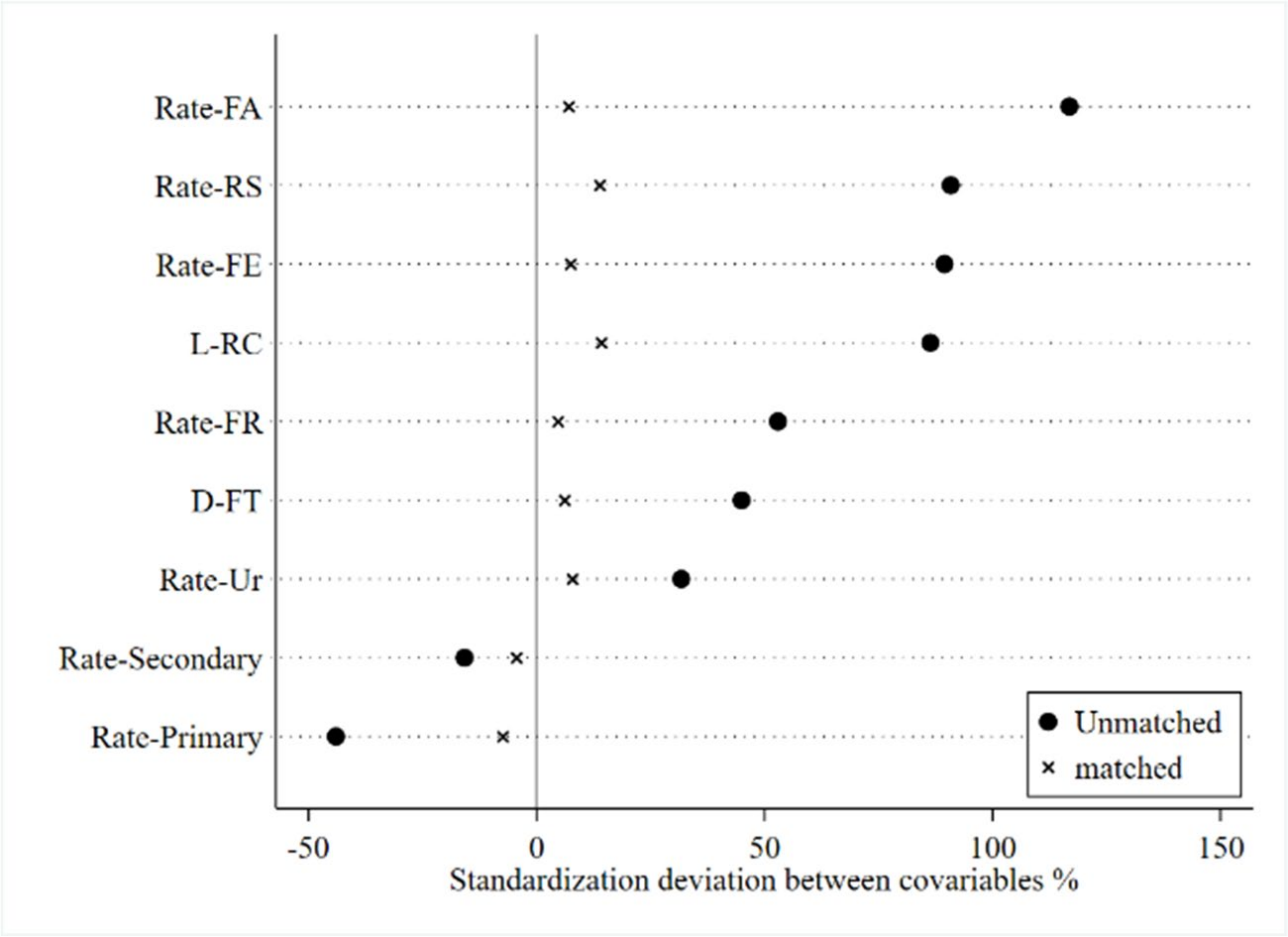
Comparison before and after Mahalanobis distance propensity matching

In this paper, the Logit model method was used to estimate the control variables of the model (1) to get the propensity score. Then, the Mahalanobis distance matching method was used to apply the “common support” condition for matching processing, and the results are presented in Fig. 1 and Table 2. According to the data in Table 2, after PSM treatment, the difference between the treatment group and the control group was significantly reduced, and the standard deviation of the control variables was adjusted. At the same time, most of the *P*-values of the balance test of the matched control variables increased significantly, and the null hypothesis could not be rejected, indicating that the overall sample met the requirements of the balance test. According to Fig. 1, the standardization deviation between covariables after propensity matching tends to converge, indicating that the suitability of matching has been proven. Therefore, the matching method adopted in this paper is reasonable on the whole, which further verifies the relative effectiveness of the matched data and makes the matching results more balanced and reliable. Based on this, the matched data can lay a foundation for further analysis of multi-phase DID.

**Table 2:** Comparison before and after Mahalanobis distance propensity matching

	Unmatched	Mean		Deviation correction		*t*-test	
Control variable	Matched	Treatment group	Control group	Deviation	Deviation reduction %	*t*	*P*
Rate-Ur	U	0.34425	0.15502	31.7		4.69	0.000
	M	0.34425	0.29761	7.8	75.4	3.95	0.000
Rate-primary	U	0.38044	0.4056	− 15.8		− 3.25	0.001
	M	0.38044	0.3875	− 4.4	72.0	− 0.64	0.524
Rate-secondary	U	0.2127	0.2635	− 44		− 7.84	0.000
	M	0.2127	0.22129	− 7.4	83.1	− 1.32	0.188
Rate-FR	U	1.0088	0.66639	90.8		20.80	0.000
	M	1.0088	0.95663	13.8	84.8	1.87	0.062
Rate-FE	U	0.9445	0.54159	86.4		23.82	0.000
	M	0.9445	0.87829	14.2	83.6	1.76	0.079
D-FT	U	0.06932	0.04592	52.9		16.87	0.000
	M	0.06932	0.06727	4.7	91.2	0.58	0.560
Rate-RS	U	0.52795	0.20942	89.5		27.80	0.000
	M	0.52795	0.50141	7.5	91.7	0.87	0.384
L-RC	U	8.9949	5.4131	44.7		9.92	0.000
	M	8.9949	8.5077	6.1	86.4	0.84	0.400
Rate-FA	U	1.2626	0.58756	116.7		28.22	0.000
	M	1.2626	1.22	7.0	94.0	0.88	0.378

### Visual analysis of DID

**Fig. 2:**
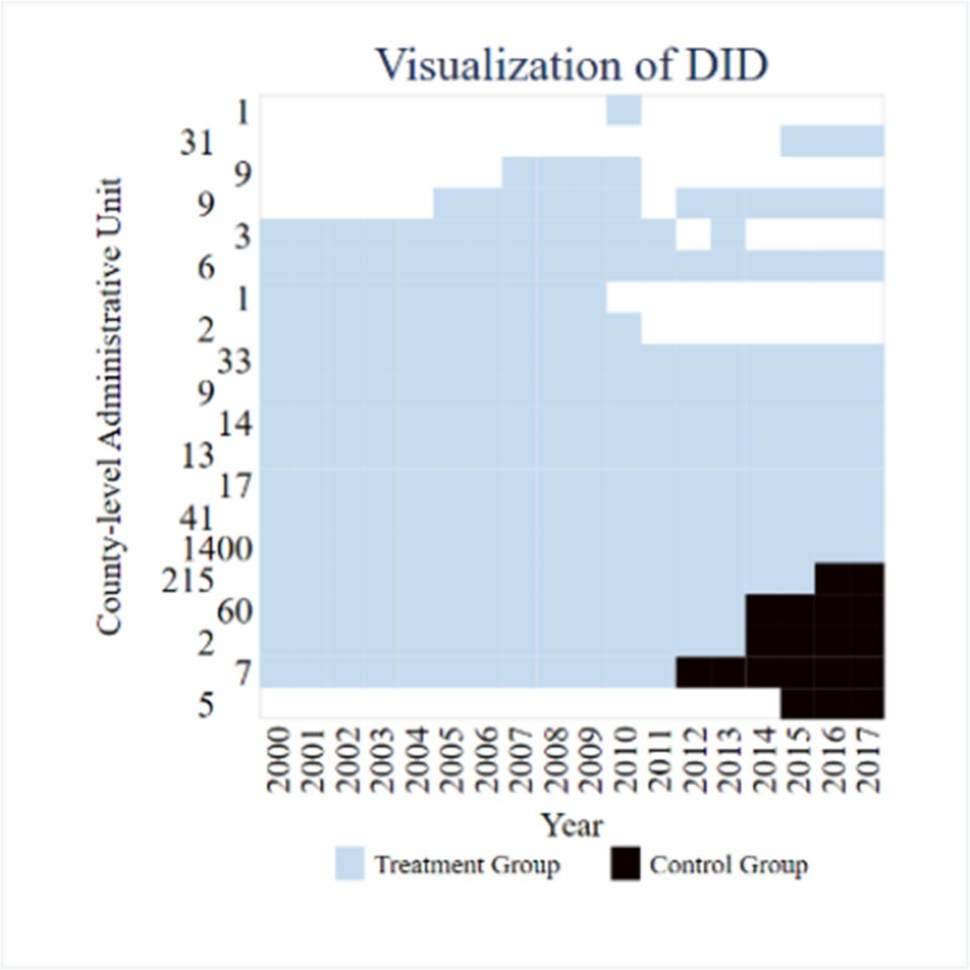
Visualization of DID

In view of the impact of clean energy demonstration provincial policies on carbon intensity in China, the visualization analysis of DID analysis panel data based on PSM matching is shown in Fig. 2. The comparison between the policy landing time of clean energy demonstration provinces and the number of control groups is reasonable. The data samples used in this paper can better reflect the actual situation based on the impact of clean energy demonstration provinces’ policies on carbon intensity at the county level.

### Baseline regression analysis

According to the matched samples, model (1) was used to test the average effect of clean energy demonstration provincial policies on carbon intensity in pilot counties. The control variables, time and individual fixed effects, and clustering were gradually added to the baseline regression. The regression results were shown in columns (1) to (4) of Table 3. After introducing the time and individual fixed effect to mitigate the problem of missing variables varying with individual and time, the regression coefficient is 63.04%, and the confidence interval is 99%. In this paper, column (4) was used as the baseline regression result for analysis, and it was found that after clustering to counties, the clean energy demonstration provincial policies significantly improved the carbon intensity of the pilot areas at the county level. The above results indicated that the clean energy demonstration provincial policies increased the carbon emissions per unit of GDP at the county level; hypothesis 1 is true. This is quite different from the conclusion drawn by Zhou et al. (2023) based on provincial panel data using PSM-DID that carbon emissions in pilot areas can be suppressed and economic development level can be improved at the same time (Zhou et al. 2023). This paper will further analyze the specific impact of clean energy demonstration provincial policies on carbon emissions and economic development based on county panel data.

**Table 3:** Baseline regression

	(1)	(2)	(3)	(4)
Control variable	*CI*	*CI*	*CI*	*CI*
*Policy*	− 1.5138**	− 2.7787***	0.6304***	0.6304***
	(0.6980)	(0.1817)	(0.0856)	(0.2181)
Rate-Ur		0.1953***	0.0403***	0.0403
		(0.0308)	(0.0133)	(0.0429)
Rate-primary		4.2275***	− 1.1838***	− 1.1838**
		(0.3014)	(0.1856)	(0.5404)
Rate-secondary		1.8020***	1.2582***	1.2582*
		(0.3474)	(0.2549)	(0.7343)
Rate-FR		3.7839***	3.6896***	3.6896***
		(0.0918)	(0.0697)	(0.4468)
Rate-FE		0.7889***	0.6906***	0.6906***
		(0.0852)	(0.0499)	(0.2009)
D-FT		18.7580***	1.6839***	1.6839
		(1.1561)	(0.5933)	(2.1884)
Rate-RS		1.0103***	0.3999***	0.3999
		(0.2144)	(0.1090)	(0.3375)
L-RC		0.0318***	0.0018	0.0018
		(0.0061)	(0.0029)	(0.0073)
Rate-FA		− 0.9182***	0.0294	0.0294
		(0.0662)	(0.0332)	(0.0821)
Observation	32,492	20,119	20,094	20,094
*R*-squared	0.000	0.160	0.901	0.901
Time fixed effects			Yes	Yes
Individual fixed effects			Yes	Yes

1. *, **, and *** denote significant at the level of 10%, 5%, and 1%, respectively, which will not be repeated below

### Parallel trend test

In order to test whether the growth trend of carbon intensity occurred before the implementation of clean energy demonstration provincial policies, this paper uses the event study method to test the parallel trend hypothesis involved in baseline regression. The specific test model is shown as follows:

4 $${CI}_{i,t}={\alpha }_{0}+{\sum }_{k\ge -7\prime}^{{3}{\prime}}{\varpi }_{k}{D}_{i,t0+k}+{\alpha }_{1}\times {Policy}_{i,t}+\gamma \times {Control}_{i,t}+{\lambda }_{1}+{\mu }_{t}+{\varepsilon }_{i,t}$$

In Formula (4), ${D}_{i,t0+k}$ represents dummy variable, ${t}_{0}$ represents the year when the clean energy demonstration province’ s policies are implemented, *k* represents the year before and after the implementation of the clean energy demonstration province’ s policies, *k* = − 7′, − 6, − 5, − 4, − 3, − 2, − 1, 0, 1, 2, 3′, where − 7′ represents the seventh year and earlier years before the implementation of the clean energy demonstration provincial policy as the baseline group, and 3′ represents the third year and later years after the implementation of the clean energy demonstration provincial policy as the baseline group. The core of the event study test is whether ${\varpi }_{k}$ is significantly different from 0 before and after the implementation of the clean energy demonstration provincial policy. As shown in Table 4, carbon intensity based on panel data at county level did not show significant and robust continuous positive correlation before the implementation of clean energy demonstration province policies. There was no time lag in the impact of policies on carbon intensity at county level, and the impact effects gradually increased with the extension of time.

**Table 4:** Parallel trend test

Relationship with policy implementation time	*CI*	Standard error
6 year before implementation	− 0.1061	(0.0900)
5 year before implementation	0.1037	(0.1179)
4 year before implementation	0.0686	(0.1341)
3 year before implementation	0.1319	(0.1529)
2 year before implementation	0.2686	(0.1657)
1 year before implementation	0.3522	(0.2162)
the year of implementation	0.4234*	(0.2291)
1 year after implementation	0.8155***	(0.2923)
2 year after implementation	3.9800***	(1.3838)
3 years after implementation and subsequent years	1.6305*	(0.9177)
Control variables	Yes	
Observations	20,094	
*R*-squared	0.902	
Time fixed effects	Yes	
Individual fixed effects	Yes	

* and *** denote significant at the level of 10% and 1%, respectively

### Further analysis of carbon emissions and economic growth

Based on the regression results of China’s county panel empirical data in the benchmark regression, this paper conducted an in-depth analysis of the impact of clean energy demonstration province policies on carbon emissions and economic growth. Based on model (2) and model (3), multi-phase DID based on PSM matching was used for regression analysis, and the regression results are shown in Table 5. Columns (2) and (4) show the regression results of adding time-fixed effects and individual fixed effects and clustering them to the county level, which is used as the benchmark regression of clean energy demonstration provincial policies on carbon emissions and economic growth for in-depth analysis. In regional economy and the emissions of carbon, the regression coefficients are divided into 11.13% and 13.33% and at 1% significance level, and clean energy demonstration province policies for regional carbon emissions of regression coefficient are greater than the regression coefficient of economic positive stimulus. It is proven that from the county perspective, while significantly stimulating economic growth, the clean energy demonstration provincial policy has brought about an additional increase in carbon emissions. At present, a good development system combining economy and environment has not been established. Hypothesis 2 is valid.

**Table 5:** Carbon emissions and economic growth

	(1)	(2)	(3)	(4)
Control variable	*Y*	*Y*	*CE*	*CE*
*Policy*	0.8445***	0.1113***	0.0928*	0.1333***
	(0.0414)	(0.0154)	(0.0540)	(0.0290)
Rate-Ur	0.0142**	0.0040	0.0420***	− 0.0008
	(0.0070)	(0.0034)	(0.0091)	(0.0011)
Rate-primary	0.3464***	0.3896***	0.8142***	0.3594***
	(0.0681)	(0.0503)	(0.0895)	(0.1003)
Rate-secondary	− 2173.6469***	− 5651.0435***	4103.8923***	− 832.3637*
	(207.2981)	(334.3716)	(272.6656)	(470.2269)
Rate-FR	− 2790.5746***	− 366.8251**	− 958.3193***	723.4245**
	(190.2250)	(150.0041)	(253.1290)	(313.3739)
Rate-FE	− 2.8252***	− 0.1311	− 3.2965***	0.9683**
	(0.2620)	(0.1366)	(0.3434)	(0.4833)
D-FT	− 1.9155***	0.0807***	− 1.1593***	0.4941**
	(0.0485)	(0.0302)	(0.0637)	(0.2398)
Rate-RS	3073.0217***	98.6675	1793.3315***	320.0016
	(149.5696)	(70.2229)	(196.5814)	(267.6280)
L-RC	− 3.2333***	− 1.1320***	− 3.5228***	− 0.3178
	(0.0785)	(0.0724)	(0.1032)	(0.2253)
Rate-FA	− 0.0129***	− 0.0034***	− 0.0343***	0.0113
	(0.0014)	(0.0006)	(0.0018)	(0.0082)
Observation	20,553	20,528	20,119	20,094
*R*-squared	0.485	0.989	0.388	0.922
Time fixed effects		Yes		Yes
Individual fixed effects		Yes		Yes

*, **, and *** denote significant at the level of 10%, 5%, and 1%, respectively

Although the pilot governments of clean energy demonstration provinces have made efforts to improve the value of the industrial chain and adjust the industrial structure (Zhou et al. 2023), they have not been able to achieve the dual benefits of economy and environment according to the above empirical data regression analysis of county-level administrative units. Therefore, more feasible strategies need to be explored to promote the mutual promotion between clean energy development and economic development based on empirical data analysis based on the county level.

### Robust test

#### CSDID

Goodman-Bacon (2021) from the theory and practice perspective proves that to get an unbiased estimate of the average treatment effect based on multiple-phase difference in difference model, two-way fixed-effects estimators are also necessary to satisfy the requirement that treatment effects be constant both across groups and over time, which is often contrary to empirical experience (Goodman-Bacon 2021). Through data simulation Baker et al. (2022) finds that the biased treatment effect estimated by multi-phase DID may even be opposite to the sign of the real treatment effect (Baker et al. 2022). Callaway and Sant’anna (2021) proposed a new method for identifying heterogeneous multi-phase DID, that is, CSDID (Callaway and Sant’anna 2021). CSDID can be used to measure the group average treatment effect on the treated (group ATT): the average processing effect of the weighted sum of groups according to the time first processed. This can effectively measure the effect of error when the treatment effect does not change over time, but varies between individuals.

Based on the premise that the policy treatment effect is irreversible, the weighted ATT and group ATT of the time (G) covered by the clean energy demonstration provincial policy for the first time are shown in Table 6.

**Table 6:** CSDID regression analysis

	Correlation	Standard deviation	*P*
G-average	0.065	0.278	0.019
G-2012	− 1.924	0.584	0.001
G-2014	0.282	0.032	0.000
G-2016	0.069	0.024	0.013

The *Policy* correlation coefficient of Ningxia (G-2012), which implemented the policy in 2012, is − 192.4%, and the *P*-value is 0.1%, indicating that the clean energy demonstration provincial policy has a significant carbon intensity reduction effect on the counties included in Ningxia Province. Ningxia Province is rich in energy resources such as coal, natural gas, and wind energy. With the support of policies, Ningxia Province has vigorously developed new energy industries, especially solar and wind energy industries. Ningxia has built large-scale solar power and wind power farms, becoming one of China’s important new energy bases. Ningxia actively develops high-tech industries, including electronic information, new materials, bio-medicine, modern equipment manufacturing, and other fields. The Ningxia government has launched a large number of deployments around promoting scientific and technological innovation and industrial transformation and upgrading, attracting a number of high-tech enterprises and research institutions to land in Ningxia, so the clean energy demonstration provincial policy has better policy performance in the county-level administrative units of Ningxia Province.

For Zhejiang Province (G-2014), where the policy was implemented in 2014, the correlation coefficient of *Policy* counties included was 28.2%, which was significant at 99% confidence interval. For Sichuan and Gansu provinces (G-2016) where the policy was implemented in 2016, the county-level *Policy* correlation coefficient was 6.9%, and the *P* value was 1.3%. For Zhejiang, Sichuan, and Gansu provinces, the clean energy demonstration provincial policy has led to an increase in carbon intensity, failing to balance economic development with energy conservation and emission reduction.

In general, based on the analysis of CSDID method, the average treatment effect was 6.5%, and the *P*-value was 1.9%, which was consistent with the baseline regression. This confirms the robustness of baseline regression, that is, from the county dimension as a whole, clean energy demonstration provinces have improved carbon intensity and failed to achieve effective unity of economic development and green and low carbon.

### Change the propensity matching method

In this paper, kernel matching, caliper (radius) matching, and nearest neighbor matching are used as the criteria for bias matching to conduct multi-stage differential regression analysis to investigate the robustness of reference regression. The results are shown in columns (1) to (3) of Table 7. The regression results are all at the 1% significance level, and the regression coefficients are fluctuated between 50.4 and 54.12%, which is consistent with the results of baseline regression, further proving the robustness of baseline regression.

**Table 7:** Robustness test PSM-DID

	(1)	(2)	(3)
Control variable	*CI*	*CI*	*CI*
*Policy*	0.5412***	0.5040**	0.5040**
	− 0.204	(0.2009)	− 0.2009
Rate-Ur	0.5738	0.7958*	0.7958*
	− 0.4895	(0.4572)	− 0.4572
Rate-primary	− 3.0132***	− 3.1876***	− 3.1876***
	− 1.1508	(1.1685)	− 1.1685
Rate-secondary	0.0608	0.0427	0.0427
	− 1.6651	(1.7007)	− 1.7007
Rate-FR	3.9331***	3.8317***	3.8317***
	− 0.7391	(0.7205)	− 0.7205
Rate-FE	0.4670**	0.4861**	0.4861**
	− 0.2196	(0.2192)	− 0.2192
D-FT	2.6567	2.6694	2.6694
	− 2.6718	(2.6675)	− 2.6675
Rate-RS	0.4824	0.4748	0.4748
	− 0.5172	(0.5281)	− 0.5281
L-RC	− 0.0064	− 0.0079	− 0.01
	− 0.0126	(0.0132)	− 0.0132
Rate-FA	− 0.0377	− 0.0278	− 0.0278
	− 0.1095	(0.1110)	− 0.111
Observation	7983	7918	7918
*R*-squared	0.910	0.911	0.911
Time fixed effects	Yes	Yes	Yes
Individual fixed effects	Yes	Yes	Yes

*, **, and *** denote significant at the level of 10%, 5%, and 1%, respectively

### Placebo test

**Fig. 3:**
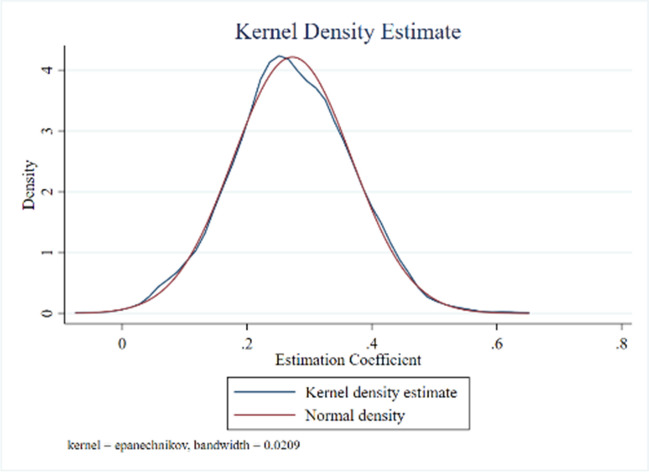
Time placebo

**Fig. 4:**
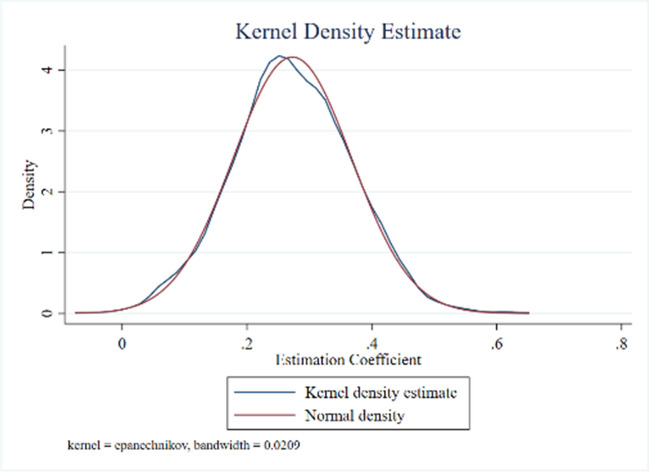
Individual placebo

(1) Time placebo. Referring to the practice of Cao (2020), the policy time point in the policy coverage area was randomly advanced and repeated 1000 times (Cao 2020). The results are shown in Fig. 3, the regression coefficient of clean energy demonstration province policies on carbon intensity meets the normal distribution on the whole, and the average value is far less than 63.04% of the estimated true coefficient of the benchmark regression, that is, the enhancement effect of the random advance of the implementation time of clean energy demonstration province policies on carbon intensity decreases significantly. That is to say, the random advance of the implementation time of clean energy demonstration provincial policies will significantly weaken the enhancement effect on carbon intensity.

(2) Individual placebo. The clean energy demonstration provincial policy coverage area in the treatment group is regarded as the new control group; in the original control group, the same number of county-level administrative districts of the original treatment group is selected as the new treatment group, and the policy implementation time and a batch of the county-level administrative districts in the new treatment group are the same as those in the original treatment group, and the above operations were repeated for 1000 times (Cao 2020). As shown in Fig. 4, the regression coefficient of the policies of clean energy demonstration provinces is close to the normal distribution, and the mean value is close to 0, satisfying the normal distribution trend on the whole.

Both time and individual placebo demonstrated the robustness of baseline regression from a counterfactual perspective.

### Mechanism test

Based on the benchmark regression result of the policy effect of China’s clean energy demonstration province on the increase of carbon intensity at the county level, this paper explores the mechanism.

According to theoretical analysis, the influence of clean energy demonstration province policies on carbon intensity mainly affects regional carbon intensity through technological progress and industrial-added value. In this paper, technological progress (*Tech*) and the ratio of industrial added value to GDP (*Ratio*) are selected as mediating variables. As for the indicators of technological progress in mainstream literature, the numbers of patent applications, authorization, and R&D investment are mostly used (Dong and Li 2020; Rahman et al. 2022). In this paper, the sum of the number of patent invention publications and authorization is used as the mediated variable. According to the production method, industrial-added value is the difference between the total industrial output and various intermediate inputs (Dong and Li 2020). As the final result of production activities, industrial value added not only reflects the production activities and economic benefits of enterprises but also indirectly reflects the types of asset investment of enterprises, that is, strategic investment or tactical investment. Therefore, this paper chooses the proportion of industrial-added value in GDP to represent the upgrading of industrialization.

5 $${Tech}_{i,t}={\alpha }_{0}+{\alpha }_{2}\times {Policy}_{i,t}+\gamma \times {Control}_{i,t}+{\lambda }_{1}+{\mu }_{t}+{\varepsilon }_{i,t}$$

6 $${Ratio}_{i,t}={\alpha }_{0}+{\alpha }_{3}\times {Policy}_{i,t}+\gamma \times {Control}_{i,t}+{\lambda }_{1}+{\mu }_{t}+{\varepsilon }_{i,t}$$

Formulas (5) and (6), respectively, verify the mediating effect of the technological progress and the ratio of industrial added value to GDP on the carbon intensity of county administrative units by clean energy demonstration provinces. In view of the potential endogeneity of the mediating effect of the stepwise verification method, this paper uses the method of Jiang (2022) for reference, which only the role of the core explanatory variable *Policy* on the mediating variable (*Tech* and *Ratio*) is verified. If ${\alpha }_{2}$ and ${\alpha }_{3}$ in Eqs. (5) and (6) are significant, then technological progress and the ratio of industrial added value to GDP play a mediating role.

As shown in column (1) of Table 8, *Policy* has a significant restraining effect on technological progress, and the correlation coefficient is − 2271.62%, which is significant at the level of 1%, indicating that the technological level plays an important transmission mechanism in the carbon intensity of clean energy demonstration province policies. Although the provincial data regression analysis found that this policy has significantly improved the technology level, from the provincial dimension, government regulation and regulation can force the technological progress of enterprises, and technological progress plays an important driving role in the evolution of the leading industry to a higher level (Zhou et al. 2023). However, based on the empirical facts of county data, it is found that the development of county technology level under the background of the implementation of clean energy demonstration provincial policies is restrained. This may be because the county’s science and technology base is relatively weak, relatively strict government regulation, and government jurisdiction make enterprises reduce innovation efforts and tired of increasing technology investment. The excessively high cost of pollution control does not promote the self-innovation of environment-friendly technologies in counties but increases the burden on enterprises and causes them to reduce the resources devoted to technologies. Hypothesis 3 is valid.

**Table 8:** Mechanism analysis

	(1)	(2)
Control variable	*Tech*	*Ratio*
*Policy*	− 22.7162***	− 0.0676***
	(7.7703)	(0.0202)
Rate-Ur	0.4256	− 0.0054
	(0.2916)	(0.0051)
Rate-primary	− 37.5212	0.9896***
	(33.9549)	(0.0306)
Rate-secondary	23.1984	0.0238
	(16.5076)	(0.0295)
Rate-FR	− 38.2945***	0.0539
	(9.9198)	(0.0358)
Rate-FE	49.4818***	− 0.0156**
	(8.4249)	(0.0067)
D-FT	704.0274***	0.5508***
	(172.8687)	(0.2132)
Rate-RS	− 116.4877***	− 0.0280
	(23.3486)	(0.0179)
L-RC	2.3500***	0.0003
	(0.6144)	(0.0003)
Rate-FA	− 22.0485***	− 0.0164***
	(5.3396)	(0.0052)
Observation	17,380	11,740
*R*-squared	0.393	0.919
Time fixed effects	Yes	Yes
Individual fixed effects	Yes	Yes

** and *** denote significant at the level of 5% and 1%, respectively

As shown in column (2) of Table 8, *Policy* has a significant damping effect on the ratio of industrial added value to GDP, with a correlation coefficient of − 6.76%, which is significant at the level of 1%. *Policy* inhibits green consumption transition by reducing the ratio of industrial added value to GDP and ultimately increases carbon intensity in counties. That may be because the county’s rougher industries are being forced to shift to cleaner and greener methods of production as environmental policies tighten. However, limited capital and relatively poor management ability increase the production cost of these county industries, squeezing the original resource space of input, scale economy, and higher production capacity, thus reducing the industrial-added value and production enthusiasm. Hypothesis 4 is valid.

From an overall perspective, the overall impact of clean energy model provincial policies on carbon intensity is complex. It should be pointed out that the comprehensive effect of policies on county carbon intensity will depend on the combined effects of policies on technological level containment and industrial added value reduction. In this paper, it is ultimately reflected in the increase of carbon emissions per unit of GDP.

### Heterogeneity analysis

Considering China’s vast territory and significant heterogeneity between regions (Razzaq et al. 2023), this paper divided the whole sample into two sub-samples according to the Hu Line (or Heihe-Tengchong Line or Aihui-Tengchong Line) (Dong and Li 2020) to further explore the heterogeneity of policy implementation effects caused by different geographical locations and resource endowments. The whole sample is divided into two sub-samples: west of the Hu Line and east of the Hu Line. The Hu Line is a comparison line for the population density of China proposed by the Chinese geographer Hu Huanyong (1901–1998) in 1935. Besides, Hu Line coincides with the transition area of China’s summer monsoon. It is not only the dividing line of China’s climate and environment but also the dividing line of China’s population and economic development level, as well as social pattern. Concretely speaking, 94% of China’s population is distributed on the east side of the line, while only 6% of the population is distributed on the west side, and the economic level gap between the two sides of the line is very significant. The regression results are shown in columns (1) and (2) in Table 9.

**Table 9:** Heterogeneity analysis

	(1)	(2)
Control variable	*CI*	*CI*
*Policy*	3.2047***	1.1960***
	(1.1734)	(0.1528)
Rate-Ur	− 0.3934	0.0392
	(1.4140)	(0.0436)
Rate-primary	2.2547	− 1.5323***
	(2.6962)	(0.4674)
Rate-secondary	5.2314	0.6748
	(3.9279)	(0.6942)
Rate-FR	0.9820**	4.0639***
	(0.4036)	(0.4813)
Rate-FE	0.3219	0.8084***
	(0.4181)	(0.2267)
D-FT	− 0.4104	− 0.6920
	(2.1552)	(2.7271)
Rate-RS	0.8114	0.5638
	(0.7118)	(0.3963)
L-RC	− 0.0066	− 0.0016
	(0.0152)	(0.0089)
Rate-FA	0.0925	− 0.0414
	(0.1023)	(0.0908)
Observation	1328	18,759
*R*-squared	0.876	0.917
Time fixed effects	Yes	Yes
Individual fixed effects	Yes	Yes

** and *** denote significant at the level of 5% and 1%, respectively

Although, the regression coefficients of *Policy* in the two sub-samples are positive in general and significant at the 1% level. However, in the east of the Hu Line, the positive improvement effect of the clean energy demonstration provincial policy on county carbon intensity is 119.60%, which was significantly lower than the 320.47% in the west of the Hu Line. Overall, for counties east of the Hu Line, although the clean energy demonstration provincial policies still have a significant impact on the improvement of carbon intensity, it is more possible for these regions to achieve high-quality development with both economy and environment as a whole, compared with counties west of the Hu Line.

The regional policy effect gap between east and west of Hu Line is caused by many reasons. The region east of the Hu Line has basically left behind the stage of development lag, and its comprehensive strength and interaction between urban and rural areas have been enhanced and improved to a certain extent. While, the west of the Hu Line has not completely reversed the underdevelopment pattern, which means the inter-regional gap is further widen under the background of rapid growth. All these will further affect the local level of science and technology, the degree of urbanization, the ratio of industrial-added value to GDP, and the efficiency of resource allocation which plays critical role in the resilience of economy (Wu et al. 2023). Good economic resilience, strong industrial base, and healthy industrial structure will undoubtedly amplify the policy effect of clean energy demonstration provinces and better promote low-carbon transformation and sustainable development.

The areas to the east of Hu Line are mostly located in China’s coastal areas and have a high degree of international integration. This has made it easier for the region to attract foreign investment and technology introduction, facilitating the flow of innovative resources and the exchange of knowledge. At the same time, these regions are usually among the most economically developed and open regions in China, with high economic strength and innovation potential, providing favorable conditions for innovation. In addition, the area east of Hu Line has many well-known higher education institutions and research institutions, which have trained a large number of high-quality talents and promoted the development of scientific research and technological innovation. Innovation plays a crucial role in reducing carbon intensity, which will directly affect the policy effect performance of clean energy demonstration provinces.

In addition, the area to the east of the Hu Line has abundant resources compared to the west area, such as human resources, financial resources, technical resources, policy resources, and consumer markets. This is similar to the resource gap between urban and non-urban areas, where resources are more abundant; it is more conducive to the green development of the economy, and economy and society as a whole. The above reasons will also lead to these regions can better gradually reverse the growth trend of carbon intensity, and better achieve future economic and environmentally friendly development.

## Conclusion

Based on China’s county-level unbalanced panel data from 2000 to 2020, this paper uses the PSM DID method to explore the impact of clean energy demonstration provincial policies on county carbon intensity and its mechanism. This paper finds the following conclusions. First, the clean energy demonstration provincial policies can significantly increase the carbon intensity of the counties covered by the policies, to be specific, which promote the economic development in pilot counties and increase carbon emission at the same time. Second, there is a mediating mechanism of technological progress and the ratio of industrial added value to GDP in increasing carbon intensity at county level for clean energy demonstration province policies, in detail, the curb of technological progress and the reduction of industrial added value both result in county level carbon intensity increasing. Thirdly, the heterogeneity analysis found that the promotion effect of clean energy demonstration provincial policies on the carbon intensity of counties east of the Hu Line was significantly weaker than that of the counties west of the Hu Line because of the rich resources, better industrial base, and level of technology; all these make it greater possibilities for achieving both economic and environmental development in the future.

Based on the above analysis, this paper draws the following policy implications. First, while paying attention to economic development, we should pay more attention to county-level carbon emissions and introduce more specific green development policies for counties. Focusing only on provincial or municipal policy formulation or data performance, it is easy to ignore the indispensable role of county-level administrative units in energy conservation and emission reduction. Second, the government should guide county-level administrative units to improve their industrial structure and industrial base through major projects and financial subsidies. Technological progress and upgrading of industrialization play a significant mediating effect on the policy effect of clean energy demonstration province to improve county carbon intensity. Technological progress and upgrading of industrialization can better promote the “power engine” of green and high-quality development of county economy, reduce carbon intensity, and promote high-quality development of county economy at the same time. We will make greater use of the policy effects of green development policies, such as clean energy demonstration provinces. Third, taking into account the differences in industrial resource endowments in different regions of China, more precise regional development policies should be formulated according to local conditions. For example, for non-urban county-level administrative units and areas west of the Hu Line, based on the scarcity of technical resources, human resources, financial resources, and market resources, in addition to further introducing policies and regulations with strong practicality and promoting green economy, attention should be paid to the scarcity of resources themselves, and supporting measures should supplement the resources necessary for the development of these areas.

The research in this paper is not only applicable to China but also to many developing countries whose economies are in urgent need of green and sustainable transformation. In addition, the study of this paper also provides some enlightenment for these countries with multi-level administrative bodies on how to formulate development policies that take into account the relatively micro and relatively macro administrative dimensions. In particular, how to enhance the effects of green policies like clean energy demonstration province policies at the county level while keeping the economy growing by technological progress and industrial upgradation. Due to the availability of data, the data in this paper only covers the data of China from 2000 to 2020 and the overall carbon emission data of counties, rather than the carbon dioxide emission data of county-level administrative units that can be traced back to specific industries or sources. In the future, more research can be carried out through more advanced measurement methods or updated and comprehensive data, and the research subject can be expanded to other countries or promoted to cross-country and regional comparisons.

## Data Availability

The data that support the findings of this study are available from the corresponding author, Lei Chen, upon reasonable request.

## Appendix 1

**Table 10:** Policy source data

Province	Policy source
Ningxia	“Letter on agreeing to carry out the construction of New Energy Comprehensive Demonstration Zone in Ningxia” issued by the National Energy Administration in July 2012
Zhejiang	Several Opinions of the National Energy Administration on Supporting the Establishment of a Clean Energy Demonstration Province in Zhejiang Province (Guoneng Xinneng (2014) No. 570) issued by issued by the National Energy Administration in 2014
Sichuan	“The Implementation Plan for Sichuan Province to create a National Clean Energy Demonstration Province” issued by the Sichuan provincial government in 2016 and approved by the National Energy Administration of China “China’ s 13th Five-Year Plan for National Energy Development” issued by the National Energy Administration of China
Tibet	“China’ s 13th Five-Year Plan for National Energy Development” issued by the National Energy Administration of China
Gansu	“National Energy Administration on supporting the establishment of new energy Comprehensive Demonstration Zone in Gansu Province” (Guoneng Xinneng (2016) No. 227) by Gansu Provincial government
Qinghai	“The Implementation Plan for Establishing a National Clean Energy Demonstration Province in Qinghai Province” issued by the Qinghai Provincial government “Reply Letter of the National Energy Administration on Matters related to the establishment of a National Clean Energy Demonstration Province in Qinghai” (National Energy Letter Xinneng (2018) No. 25) issued by the National Energy Administration of China

The policies for these clean energy demonstration provinces come from the National Energy Administration of China (NEA) and the provincial policies designated by each provincial government around China’s 13th Five-Year Plan (2016-2020) and are approved by NEA

10
